# Bergapten alleviates osteoarthritis by regulating the ANP32A/ATM signaling pathway

**DOI:** 10.1002/2211-5463.12648

**Published:** 2019-05-23

**Authors:** Yi He, Zeng Zisan, Zhenhui Lu, Li Zheng, Jinmin Zhao

**Affiliations:** ^1^ Guangxi Engineering Center in Biomedical Material for Tissue and Organ Regeneration The First Affiliated Hospital of Guangxi Medical University Nanning China; ^2^ Guangxi Collaborative Innovation Center for Biomedicine The First Affiliated Hospital of Guangxi Medical University Nanning China; ^3^ Department of Radiology The First Affiliated Hospital of Guangxi Medical University Nanning China; ^4^ Guangxi Key Laboratory of Regenerative Medicine The First Affiliated Hospital of Guangxi Medical University Nanning China; ^5^ Department of Orthopaedics Trauma and Hand Surgery The First Affiliated Hospital of Guangxi Medical University Nanning China

**Keywords:** ANP32A/ATM signal, bergapten, chondrocytes, IL‐1β, osteoarthritis

## Abstract

Osteoarthritis (OA) is a chronic degenerative disease that commonly affects the elderly. Current drug therapies for treating OA may cause adverse side effects, and so there remains a need to develop alternative treatments. Bergapten (BG) is a coumarin phytohormone that is widely found in fruits and has antioxidative and anti‐inflammatory effects. Here, we tested the hypothesis that BG may restrict the progression of OA by examining its effect on OA chondrocytes. We observed that BG significantly ameliorated interleukin (IL)‐1β‐induced expression of inflammatory cytokines and mediators, including interleukin 1 (*Il‐1*), interleukin 6 (*Il‐6*), tumor necrosis factor α (*Tnf‐α*), cyclooxygenase 2 (*Cox‐2*) and matrix metalloproteinase 13 (*Mmp‐13*), maintained chondrocyte phenotype, and promoted the secretion of cartilage‐specific extracellular matrix. We provide evidence that BG exerts its anti‐inflammatory effect by activating the ANP32A/ATM signaling pathway, which was recently verified to be associated with OA. In conclusion, these findings indicate that BG may be a potential candidate for treatment of OA.

**Abbreviations**
ANP32A: acidic leucine‐rich nuclear phosphoprotein‐32A
ATM: ataxia‐telangiectasia mutated
BG: bergapten
COX‐2: cyclooxygenase 2
DMMB: 1,9‑dimethylmethylene blue
FDA: fluorescein diacetate
GAG: glycosaminoglycan
GAPDH: glyceraldehyde 3‐phosphate dehydrogenase
H&E: hematoxylin and eosin
IL: interleukin
LPS: lipopolysaccharide
MMP‐13: matrix metalloproteinase 13
OA: osteoarthritis
PI: propidium iodide
TNF‐α: tumor necrosis factor α

Osteoarthritis (OA) is a degenerative joint disease of the elderly which seriously affects the quality of life of patients and imposes a huge economic burden on families and society 1, 2. Clinically, chronic joint pain, stiffness, bone hypertrophy, bony friction and activity limitation are the most common pathogenesis of arthritis 3, 4. Drug therapy is among the most typical choices for treating OA 5. First‐line therapies include topical non‐steroidal anti‐inflammatory drugs and oral paracetamol, which have been demonstrated to relieve pain and the inflammatory processes of OA. However, they are accompanied by adverse events and unexpected risks, such as gastrointestinal and cardiovascular complications, limiting their clinical application 6, 7.

Agents derived from plant extracts have attracted wide attention for treating the inflammatory diseases because of their multi‐target effects and minimal side effects 8, 9. Bergapten (BG; 5‐methoxypsoralen), a coumarin phytohormone that exists in multiple fruits such as figs and bergamot 10, 11, has been reported to have potential in the treatment of skin diseases (such as psoriasis) 12, 13. The anti‐inflammatory activities of BG, which has nearly no side effects, have been demonstrated both *in vitro* and *in vivo*. BG possibly modulated excessive cystic fibrosis lung inflammation by regulating the expression of the interlukin (IL)‐8 gene in bronchial epithelial cells 13. Bose *et al*. 14 also described that the production of pro‐inflammatory cytokines, such as tumor necrosis factor α (TNF‐α) and IL‐6, could be significantly restrained by BG in lipopolysaccharide (LPS)‐stimulated human peripheral blood mononuclear cells. Besides, BG has been proven to suppress inflammation by inhibiting the recruitment of neutrophils and macrophages in a tail‐cutting‐induced zebrafish injury site and reducing reactive oxygen species and nitric oxide generation 15. Furthermore, Zheng *et al*. 16 found that BG inhibited osteoclastogenesis and bone resorption stimulated by LPS, which suggests BG could be taken as a possible treatment of inflammatory bone‐destructive diseases. Thus, BG may be a potential candidate for OA therapy.

The acidic leucine‐rich nuclear phosphoprotein‐32A (ANP32A)/ataxia‐telangiectasia mutated (ATM) axis plays an important role in the process of OA. ANP32A is one of the members of the ANP32 family 17, which participate in hormone receptor interactions, enzyme inhibition, cell adhesion, cell transport, gene expression regulation and apoptotic signaling pathways 18, 19, 20. Single‐nucleotide polymorphisms in the ANP32A gene were genotyped in two independent UK cohorts and a cohort from the Netherlands (a total of 2170 OA patients and 2849 normal controls). It was revealed that ANP32A was highly associated with OA in women 21. Cornelis *et al*. 22 identified that ANP32A alleviated the progression of OA and protected cartilage from destruction due to the positive transcriptional regulation of ATM based on microarray profiling. Therefore, ANP32A/ATM may be a potential therapeutic target of OA.

In the present research, we investigated the effect of BG on the expression of pro‐inflammatory cytokines in IL‐1β‐stimulated chondrocytes. We further studied whether BG suppresses the inflammatory factor through regulation of the ANP32A/ATM axis.

## Materials and methods

### Cell isolation and culture

All animal experimental processes were approved by the Animal Experimental Center of Guangxi Medical University (Nanning, China). Chondrocytes were isolated from the knee joints of 7‐day‐old Sprague–Dawley rats. Briefly, rats were sacrificed by an overdose of pentobarbital and the articular cartilage was collected from the knee joints. Subsequently, cartilages were cut into pieces ranging from 1 to 3 mm^3^ and digested with 2 mg·mL^−1^ collagenase II (Sigma, St. Louis, MO, USA) for 3 h at 37 °C. Then collected chondrocytes were suspended in complete culture medium supplemented with 10% fetal bovine serum (FBS; Gibco, Grand Island, NY, USA) and 1% penicillin–streptomycin (Solarbio, Beijing, China) and incubated at 37 °C in a humidified atmosphere containing 5% CO_2_.

### Bergapten treatment

Bergapten was purchased from Solarbio with purity ≥ 98% (HPLC). Second generation chondrocytes were digested and seeded into six‐well plates, 24‐well plates and 96‐well plates at a density of 5 × 10^4^, 2 × 10^4^ and 5 × 10^3^ cells/well, respectively. The experiments were divided into three groups: (a) control group (chondrocytes treated with vehicle only); (b) IL‐1β group (chondrocytes stimulated with 10 ng·mL^−1^); (c) IL‐1β + BG group (chondrocytes cultured with 10 μm of BG for 1 h then stimulated with 10 ng·mL^−1^ IL‑1β for 24 h).

### Cytotoxicity assay

To assess the cytotoxic effect of BG on chondrocytes, a Cell Counting Kit‐8 (CCK‐8; Dojindo, Kumamoto, Japan) assay was performed. Chondrocytes seeded in 96‐well plates were treated with BG at concentrations of 1, 5, 10, 50 and 100 μm for 72 h and then 10% of the volume of CCK‐8 solution was added into each well. The absorbance was measured at 450 nm by a microplate reader (Thermo Fisher Scientific, Waltham, MA, USA) after 3 h of incubation.

### Cell viability

The effect of BG on the viability of IL‐1β‐induced chondrocytes was assessed by fluorescein diacetate (FDA; Solarbio)/propidium iodide (PI; Sigma) staining. Cells were washed with PBS three times and incubated with 200 μL FDA/PI solution containing 0.5‰ FDA and 2‰ PI in the dark for 5 min at 37 °C. Images were obtained using a fluorescence inversion microscope (Leica, Wetzlar, Germany).

### Cell morphology

Cells were rinsed three times with PBS and fixed with 95% ethanol for 30 min. They were then washed by PBS again and stained with hematoxylin and eosin (H&E; Solarbio). Finally, the images were observed by an inverted microscope (Leica).

### Safranin O staining

Safranin O staining was used to assess the deposition of glycosaminoglycan (GAG). After washing three times with PBS, cells were fixed with 95% ethanol and stained with 0.1% safranin O (Solarbio) for 15 min. Images were captured through an inverted microscope.

### Cell proliferation and biochemical assay

Chondrocytes were seeded in six‐well plates and incubated with 10 μm BG for 1 h followed by stimulation with IL‐1β for 24 h. Subsequently, the cells were collected by enzyme digestion and resuspended in 1 mL PBS containing 60 μg·mL^−1^ proteinase K (Boster, Wuhan, China) and incubated at 60 °C for 12 h. After incubated with Hoechst 33258 (Boster) for 15 min, DNA content was calculated by measuring the fluorescence intensity via a fluorescence microplate reader (BioTek, Winooski, VT, USA). To evaluate the production of GAG, 1,9‐dimethylmethylene blue (DMMB; Sigma) method was used. The absorbance at 525 nm of each sample was measured by a microplate reader.

### Quantitative real‐time polymerase chain reaction analysis

Quantitative real‐time polymerase chain reaction (qRT‐PCR) was conducted to quantify the gene expression levels of OA‐specific markers including *Il‐1*,* Il‐6*,* Cox‐2*,* Tnf‐α* and *Mmp‐13* of chondrocytes. The primers used for qRT‐PCR are shown in Table 1. The total RNA was extracted from each sample with a total RNA isolation kit (Megentec, Guangzhou, China). Then a reverse transcription kit (Fermentas Company, Waltham, MA, USA) was used to reverse transcribe RNA samples into cDNA. All qRT‐PCR reactions were conducted by a LightCycler 96 system (Roche, Basel, Switzerland) under the conditions of 10 min at 95 °C to denature cDNA, then 40 cycles of 10 s at 95 °C, followed by 60 s at 60 °C that hybridized the primer with the target DNA. Experiments for each gene were assayed in sextuplicate. The relative gene expression levels were calculated by using the $2^{-\Delta \Delta C_{t}}$ method to confirm the gene expression level relative to glyceraldehyde 3‐phosphate dehydrogenase (GAPDH).

**Table 1:** Primers for RT‐PCR

Gene	Forward primer	Reverse primer
*Il‐1*	5′‐GCACAGTTCCCCAACTGGTA‐3′	5′‐GGAGACTGCCCATTCTCGAC‐3′
*Il‐6*	5′‐ACAAGTCCGGAGAGGAGACT‐3′	5′‐ACAGTGCATCATCGCTGTTC‐3′
*Cox‐2*	5′‐GATGACGAGCGACTGTTCCA‐3′	5′‐CAATGTTGAAGGTGTCCGGC‐3′
*Tnf‐α*	5′‐GACCACGTAGCCGTGTTCAG‐3′	5′‐GGGCTCCACATTGCAGAGAA‐3′
*Mmp‐13*	5′‐CAGATGGGCATATCCCTCTAAGAA‐3′	5′‐CCATGACCAAATCTACAGTCCTCAC‐3′
*Gapdh*	5′‐CTATAAATTGAGCCCGCAGC‐3′	5′‐ACCAAATCCGTTGACTCCG‐3′

### Immunofluorescence analysis

The expression levels of MMP‐13, IL‐6, ATM and ANP32A proteins in chondrocytes were quantified by immunofluorescence staining. After rinsing three times with PBS, chondrocytes were permeabilized by 0.1% Triton X‐100. The cells were incubated with primary antibodies against MMP‐13, IL‐6, ATM and ANP32A (Boster; 1 : 200) at 4 °C overnight, followed by the secondary antibody–FITC anti‐rabbit IgG (Boster; 1 : 200) in the dark for 30 min at room temperature. Finally, the nuclei were stained by 4′,6‐diamidino‐2‐phenylindole for 5 min and cells were viewed using a fluorescence microscope (Olympus, Tokyo, Japan).

### Western blot analysis

Total protein was extracted using RIPA lysis buffer (Solarbio) supplemented with phenylmethylsulfonyl fluoride (Solarbio) for 60 min on ice. Protein concentration was quantified with the BCA protein assay. Equal amounts of protein (30 μg per lane) were fractionated on 5–10% SDS/PAGE gels and transferred onto PVDF membranes (Millipore, Billerica, MA, USA). After blocking in western blocking buffer (Beyotime, Beijing, China) for 2 h, the membranes were probed at 37 °C for 3 h with primary antibodies against MMP‐13, IL‐6, ATM and ANP32A antibodies (Boster; 1 : 500). Following washing with tris buffered saline tween (TBST) and incubation with secondary antibodies (Boster; 1 : 500) for 1 h, the signals were detected and visualized with an Odyssey Infrared Imaging System (LI‐COR, St Charles, MO, USA).

### Statistical analyses

All experiments in this study were performed independently six times (*n* = 6). Values are presented as mean ± SD, and spss statistics 20.0 (IBM Corp., Armonk, NY, USA) was used for data analysis via one‐way ANOVA and Tukey's test. Differences were considered as statistically significant when *P *<* *0.05.

## Results

### Cytotoxicity of bergapten on chondrocytes

**Figure 1:**
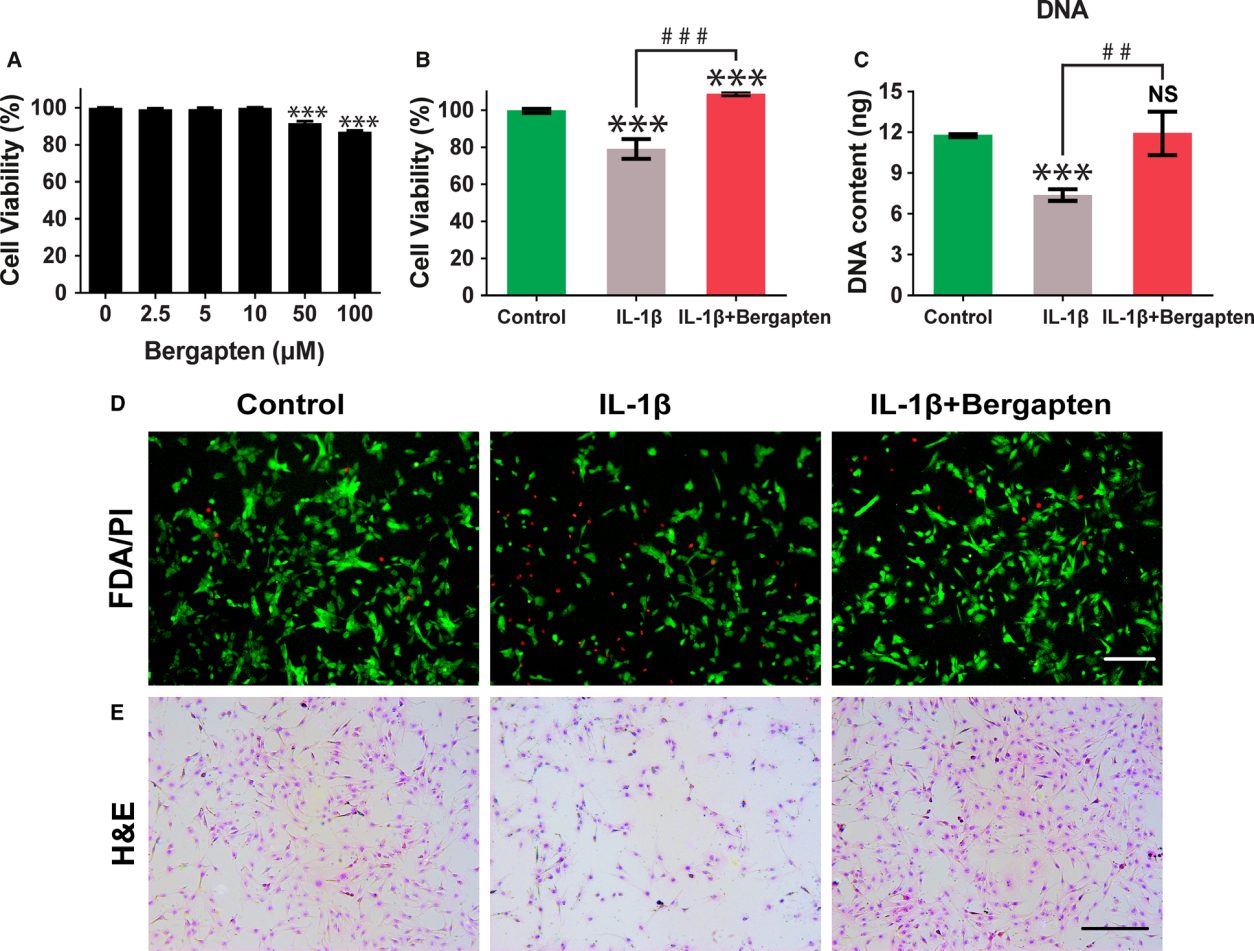
Chondro‐protective effects of BG on IL‐1β‐induced chondrocytes. (A) Cytotoxicity of BG at 0–100 μm was evaluated by CCK‐8 assay after 72 h of incubation. (B) Cell viability of chondrocytes pretreated with culture medium only or BG followed by stimulation with IL‐1β (10 ng·mL^−1^) for 24 h was assessed by CCK‐8 assay. (C) Quantification of DNA content for cell proliferation (mean ± SD,* n* = 6). (D) Cell viability by FDA/PI staining. (E) Cell morphology by hematoxylin–eosin staining (scale bars, 250 μm). Control group: chondrocytes treated with vehicle only; IL‐1β group: chondrocytes stimulated with 10 ng·mL^−1^ IL‐1β); and IL‐1β+BG group: chondrocytes cultured with 10 μm BG for 1 h then stimulated with 10 ng·mL^−1^ IL‑1β for 24 h. Mean ± SD,* n* = 6; ***P <* *0.001 *vs* the control group; ^##^ *P *<* *0.01, ^###^ *P *<* *0.001 between the indicated experimental groups; data analysis by one‐way ANOVA and Tukey's test.

To evaluate the toxicity of BG on chondrocytes, cells were treated with different doses of BG and a CCK‐8 assay was performed. The results revealed that BG at concentrations of 1–10 μm showed no cytotoxicity on chondrocytes (Fig. 1A). Thus, BG at a concentration of 10 μm was selected for the following experiments.

### Effects of bergapten on proliferation and viability of chondrocytes

The effect of BG on the viability of chondrocytes that were stimulated by IL‐1β was determined with a CCK‐8 assay. Chondrocytes were cultured with or without BG for 1 h followed by stimulation with IL‐1β (10 ng·mL^−1^) for 24 h. As demonstrated in Fig. 1B, cell viability in the IL‐1β group decreased to 79.16%. The cell viability of IL‐1β‐induced chondrocytes was increased to 108.87% with the addition of BG compared to the control group. FDA/PI staining also exhibited the same trends. More viable cells and fewer dead cells were exhibited in the BG‐treated group compare to the IL‐1β‐only group (Fig. 1D).

The proliferation of chondrocyte was investigated by detecting DNA content. Cell proliferation was significantly decreased in the treatment of IL‐1β only (*P *<* *0.05), while a higher cell proliferation level was shown with BG pretreatment as evidenced by DNA content (Fig. 1C). The results indicated that BG could effectively suppress the damage in cell proliferation induced by IL‐1β.

### Effects of bergapten on cell morphology

Morphological changes in chondrocytes after stimulation by IL‐1β were investigated by H&E staining. As shown in Fig. 1E, normal chondrocytes were polygonal in shape. However, reductions of cytoplasm, cell volume and cell number were observed in the treatment with IL‐1β. With the addition of BG, cells displayed polygonal morphology which was similar to the control. More cell colonies could be observed with the treatment by BG.

### Effect of bergapten on IL‐1β‐induced GAG loss

**Figure 2:**
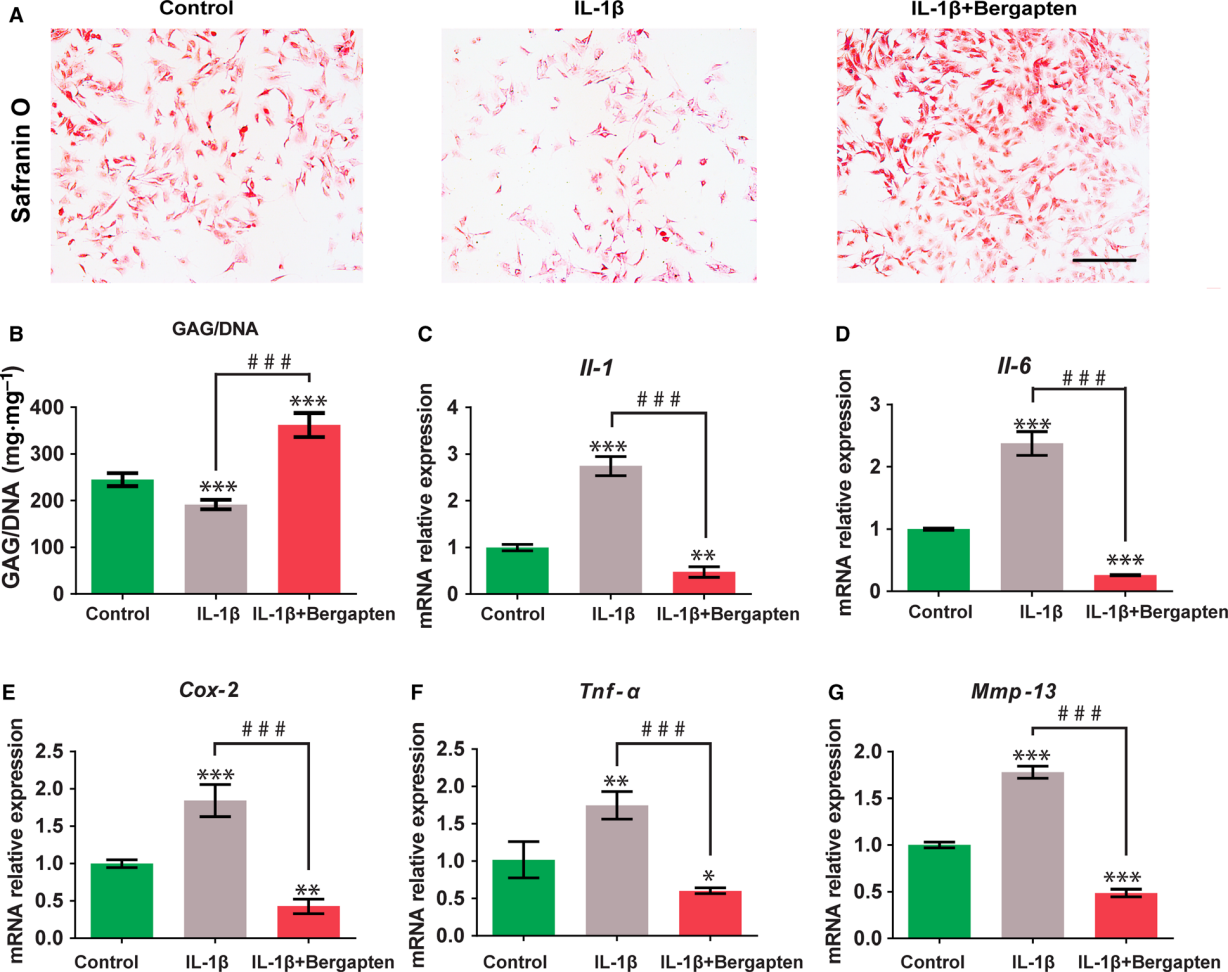
Effects of BG on IL‐1β‐induced inflammatory responses in chondrocytes: Chondrocytes were pretreated with culture medium only or BG followed by IL‐1β (10 ng·mL^−1^). (A) GAG production by Safranin O staining. Scale bars, 250 μm. (B) GAG (mg) normalized to DNA (mg). (C–G) Quantitative expression of *Il‐1* (C), *Il‐6* (D), *Tnf‐*α (E), *Cox‐2* (F) and *Mmp‐13* (G) genes detected by qRT‐PCR. Control group: chondrocytes treated with vehicle only; IL‐1β group: chondrocytes stimulated with 10 ng·mL^−1^ IL‐1β; and IL‐1β+BG group: chondrocytes cultured with 10 μm of BG for 1 h then stimulated with 10 ng·mL^−1^ IL‑1β for 24 h. Mean ± SD,* n* = 6; *P <* *0.05, **P <* *0.01, ***P <* *0.001 *vs* the control group; ^###^ *P *<* *0.001 between the indicated experimental groups; data analysis by one‐way ANOVA and Tukey's test.

The secretion of GAG in chondrocytes was determined by safranin‐O staining (Fig. 2A). Chondrocytes treated with IL‐1β showed loss of positive staining for GAG, while a large area of positive staining was exhibited in the presence of BG. The DMMB assay (Fig. 2B) showed that IL‐1β significantly decreased the production of GAG by 21.8% compared to control. Pretreating with BG increased the GAG secretion by 86.1% in comparison to the IL‐1β‐only group. This indicated that BG can restraint the IL‐1β‐induced GAG loss in chondrocytes.

### Effects of bergapten on IL‐1β‐induced inflammatory responses in chondrocytes

To determine the effects of BG on IL‐1β‐induced inflammatory responses in chondrocytes, the gene expression levels of OA‐specific markers *Il‐1*,* Il‐6*,* Tnf‐*α, *Cox‐2* and *Mmp‐13* were examined using qRT‐PCR (Fig. 2C–G). Chondrocytes stimulated with IL‐1β were found to up‐regulate the mRNA expression of OA markers compared with control group. However, cells incubated with BG reversed the IL‐1β‐stimulated upregulation of *Il‐1*,* Il‐6*,* Tnf‐*α, *Cox‐2* and *Mmp‐13* by 82.71%, 89.07%, 65.44%, 76.82% and 72.67%, respectively.

**Figure 3:**
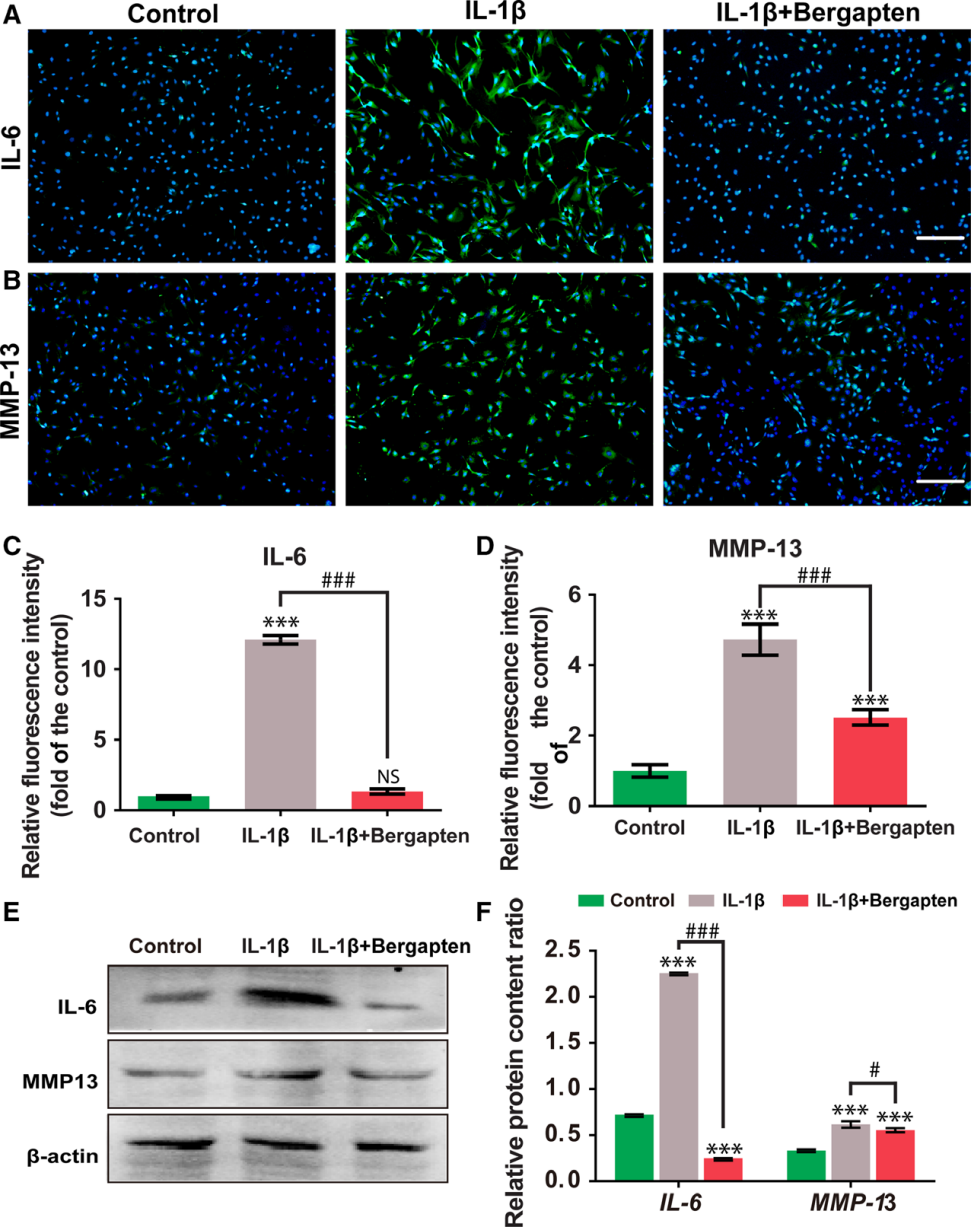
Immunofluorescence staining and western blot revealed the inhibitory effect of BG on the expression of IL‐6 and MMP‐13. (A–D) Immunostaining of IL‐6 (A) and MMP‐13 (B) and quantitative analysis of the fluorescence intensities (C,D) determined as fold‐change *vs* the control group. (E,F) Protein levels of IL‐6 and MMP‐13 determined by western blot analysis. Control group: chondrocytes treated with vehicle only; IL‐1β group: chondrocytes stimulated with 10 ng·mL^−1^ IL‐1β; and IL‐1β+BG group: chondrocytes cultured with 10 μm of BG for 1 h then stimulated with 10 ng·mL^−1^ IL‑1β for 24 h. Scale bars, 400 μm. Mean ± SD,* n* = 6; ***P <* *0.001 *vs* the control group; ^#^ *P *<* *0.05, ^###^ *P *<* *0.001 between the indicated experimental groups; data analysis via one‐way ANOVA and Tukey's test.

MMP‐13 has been proved to be a key regulator during the progression of OA 23. To explore the effect of BG on the IL‐1β‐induced upregulation of MMP‐13 in chondrocytes, the expression of MMP‐13 was analyzed by immunofluorescence staining (Fig. 3B,D) and western blot (Fig. 3E,F). As shown in Fig. 3D,F, an increased expression of MMP‐13 was displayed in the treatment with IL‐1β. However, the MMP‐13 expression was obviously suppressed by adding BG. In addition, the impact of BG on inflammatory cytokine IL‐6 was also examined by immunofluorescence staining (Fig. 3A,C) and western blot (Fig. 3E,F). BG attenuated the IL‐6 expression which was upregulated by IL‐1β.

### Effect of bergapten on ANP32A/ATM axis upon IL‐1β stimulation

**Figure 4:**
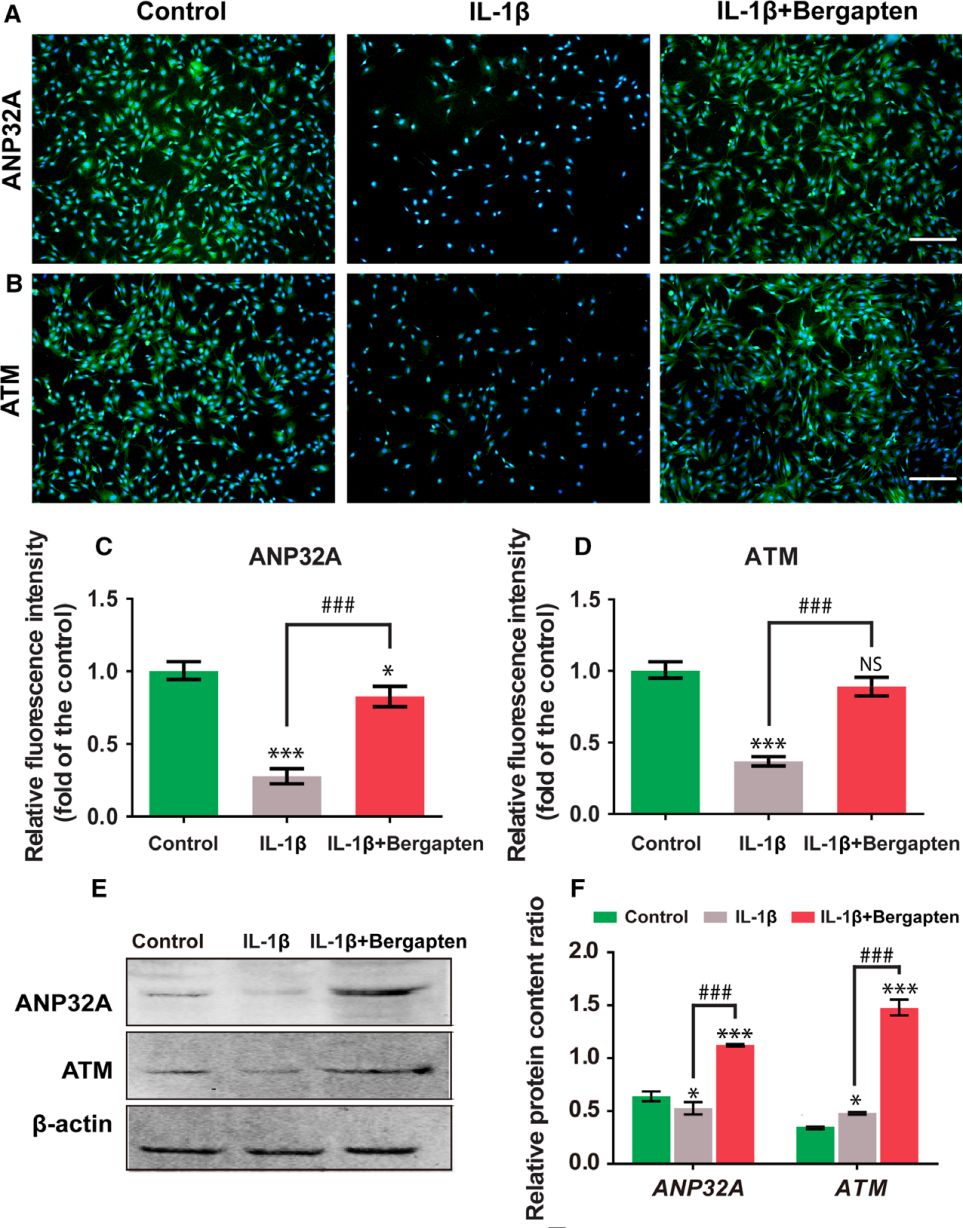
Effect of BG on ANP32A/ATM axis upon IL‐1β stimulation. (A–D) Immunostaining of ANP32A (A) and ATM (B) and relative expression (fold‐change relative to control) (C, D). (E, F) Protein levels of ANP32A and ATM determined by western blot analysis. Control group: chondrocytes treated with vehicle only; IL‐1β group: chondrocytes stimulated with 10 ng·mL^−1^ IL‐1β; and IL‐1β+BG group: chondrocytes cultured with 10 μm of BG for 1 h then stimulated with 10 ng·mL^−1^ IL‑1β for 24 h. Scale bars, 400 μm. Mean ± SD,* n* = 6; *P <* *0.05, ***P <* *0.001 *vs* the control group; ^###^ *P *<* *0.001 between the indicated experimental groups; data analysis via one‐way ANOVA and Tukey's test.

The ANP32A/ATM axis has been reported to be involved in the development of OA. The effect of BG on ANP32A/ATM signal transduction that was stimulated by IL‐1β was assessed using immunofluorescence staining (Fig. 4A–D) and western blot (Fig. 4E,F). As demonstrated in Fig. 4A–F, in the treatment of IL‐1β, the expression of ANP32A and ATM was significantly downregulated compared to control. However, a higher expression level was detected in the treatment with BG. The results indicated that BG could reverse the effect of IL‐1β in stimulating chondrocytes via the ANP32A/ATM signal.

## Discussion

This study investigated the effect of BG on OA. We found that BG could reverse IL‑1β‐induced inflammation and matrix loss of chondrocytes by regulation of the ANP32A/ATM pathway, contributing to delay in the progress of OA.

During the treatment process of OA, it is of significance to maintain the matrix metabolism of healthy cartilage and reduce cartilage degradation 24, 25. We discovered that BG constrained IL‑1β‐induced damage by accelerating the proliferation and maintaining the phenotype of chondrocytes. As evidenced by safranin O staining (Fig. 2A) and GAG content quantification (Fig. 2B), BG significantly promoted GAG secretion, which may be responsible for maintaining the balance of the extracellular matrix. MMP‐13 has been confirmed to participate in the progression of OA since it targets and degrades collagen‐II and proteoglycan, which are the main components of the extracellular matrix of cartilage 26. In this study, we demonstrated that BG inhibited IL‑1β‐induced MMP‑13 upregulation in chondrocytes (Fig. 3). In addition, BG could effectively limit the damage stimulated by IL‐1β and promote cell proliferation, which was indicated by the DNA content (Fig. 1C) and FDA/PI staining (Fig. 1D). These results demonstrated that BG may be an effective regulator in proliferation and phenotype maintenance of chondrocytes in OA.

IL‑1β can induce cellular inflammatory conditions and increase the production of pro‑inflammatory cytokines including IL‐6, TNF‐α, COX‐2 and MMP‐13, which mediate the pathogenesis of OA 27. In the previous study, BG has been reported to suppress the expression of pro‐inflammatory cytokines induced by LPS 28. Consistent with this finding, we also revealed that BG could reverse the expression of inflammatory cytokines and mediators induced by IL‑1β in chondrocytes (Fig. 2).

The regulatory effect of ANP32A in OA has been confirmed 21. A recent study also found that ATM is a positive transcriptional regulator in the expression of ANP32A 22. Furthermore, ANP32A plays an important role in the progression of OA that largely depends on positively regulating the expression of ATM 6. In our study, chondrocytes stimulated by IL‐1β decreased the expression of ANP32A and ATM, but BG reversed the effect of IL‐1β on inducing the lower expression of ANP32A and ATM (Fig. 4).

In summary, our study offers the new insight that BG can restrain the Il‐1β‐induced destruction of chondrocytes *in vitro* by inhibiting the expression of *Il‐1*,* Il‐6*,* Tnf‐α*,* Cox‐2* and *Mmp‐13*. Moreover, our results also revealed that the anti‐inflammatory reaction of BG was by activating the ANP32A/ATM axis. These findings indicated that BG may be a promising candidate for the treatment of OA.

## Conflict of interest

The authors declare no conflict of interest.

## Author contributions

JZ, LZ and ZL conceived and designed the project, LZ and YH acquired the data, ZL, ZZ and YH analysed and interpreted the data, and ZZ and YH wrote the paper.
